# 高危多发性骨髓瘤诊断与治疗中国专家共识（2024年版）

**DOI:** 10.3760/cma.j.cn121090-20240312-00090

**Published:** 2024-05

**Authors:** 中国抗癌协会血液肿瘤专业委员会骨髓瘤与浆细胞疾病学组 中国临床肿瘤学会多发性骨髓瘤专家委员会

**Keywords:** 多发性骨髓瘤, 高危, 诊断, 治疗, Multiple myeloma, High-risk, Diagnosis, Treatment

## Abstract

高危多发性骨髓瘤（High risk multiple myeloma，HRMM）是指在当前规范诊疗下，总生存期不足2～3年的骨髓瘤患者。结合多种静态和动态预后因素进行危险度分层，早期识别HRMM患者，并采用个体化风险分层治疗策略，有望显著改善HRMM患者不良生存结局。尽管近年来，国内对HRMM的临床价值已达成共识，但在HRMM的定义、高危因素、危险度分层和治疗等方面仍存在混乱和不明确之处，亟需规范。为提高中国医师对HRMM的诊治水平，中国抗癌协会血液肿瘤专业委员会和中国临床肿瘤学会多发性骨髓瘤专家委员会组织相关专家制定了本共识。该共识旨在明确HRMM的定义、高危因素和危险度分层体系，提供HRMM的治疗推荐，从而提高中国HRMM患者的生存质量和预后。

## Abstract (en)

High-risk multiple myeloma（HRMM）refers to patients with multiple myeloma whose overall survival time is less than 2-3 years under current standardized diagnosis and treatment. By combining various static and dynamic prognostic factors, risk stratification is performed to identify HRMM patients early and treat patients with personalized strategies, with the aim of significantly improving adverse survival outcomes in HRMM patients. Although the clinical value of HRMM has reached a consensus domestically in recent years, there still exist confusions and ambiguities in the definition, high-risk factors, risk stratification, and treatment of HRMM, necessitating standardization. In order to enhance the diagnostic and treatment capabilities of Chinese physicians in HRMM, the Professional Committee of Hematologic Malignancies of the Chinese Anti-Cancer Association（CACA）and the Multiple Myeloma Expert Committee of the Chinese Society of Clinical Oncology（CSCO）have organized relevant experts to develop this consensus. This consensus aims to clarify the definition of HRMM, high-risk factors, and risk stratification system, and provide treatment recommendations for HRMM, thereby improving the quality of life and prognosis of Chinese HRMM patients.

过去的二十余年，由于新治疗策略的引入，多发性骨髓瘤（MM）患者的生存期得到显著延长。但MM是一组生物学和临床高度异质性的肿瘤，仍有部分患者（15％～20％）从目前的治疗模式中获益较小[1]。这部分患者临床特征往往为侵袭性，表现为早期复发或原发难治，称之为高危多发性骨髓瘤（High risk multiple myeloma，HRMM）。临床医师应该及早识别出HRMM，给予个体化治疗。为提高中国医师对HRMM的诊断及治疗水平，中国抗癌协会（CACA）血液肿瘤专业委员会和中国临床肿瘤学会（CSCO）多发性骨髓瘤专家委员会组织相关专家经过多次讨论，制定了本版HRMM诊断与治疗中国专家共识。

一、HRMM的定义

目前缺乏对HRMM的准确定义，参考国际骨髓瘤工作组（IMWG）对HRMM定义，专家委员会认为在规范治疗的情况下，接受auto-HSCT的MM患者总生存（OS）期<3年或未接受auto-HSCT的MM患者OS期<2年定义为HRMM患者，接受auto-HSCT的MM患者OS期<2年则定义为超高危MM（Ultra-high risk MM，UHRMM）患者[2]。

二、根据预后因素识别HRMM

由于高危患者从目前标准治疗模式中获益较少，因此根据预后因素识别此类患者非常重要，其中MM肿瘤细胞的生物学特征和治疗反应是识别HRMM的决定性因素。

（一）MM的静态预后因素

1. 遗传学高危因素：细胞遗传学异常是MM危险分层体系中的核心指标，但目前对高危细胞遗传学异常（High risk cytogenetics abnormalities，HRCAs）的定义仍然存在一定的争议。2016年IMWG指南将t（4;14）、t（14;16）、t（14;20）、del（17/17p）、1q21获得/扩增、非超二倍体核型、核型del（13）、高危基因表达谱归为HRCAs[2]。在此基础上，NCCN指南2024 V1版将MYC易位、del（1p32）也归为HRCAs。如同时存在多个HRCAs，患者预后更差。Mayo诊所的mSMART 3.0指南将t（4;14）、t（14;16）、t（14;20）、del 17p、p53突变、1号染色体异常［1q21获得/扩增和del（1p）］归为HRCAs，并引入双打击和三打击MM的概念，即出现HRCAs中的任何2种或3种及以上遗传学异常[3]。

目前最常用的遗传学异常检测技术为FISH，二代测序检测到的最常见的突变（如KRAS、NRAS、DIS3、BRAF、FAM46C）对预后的影响尚不明确，只有TP53突变对预后影响较大[4]。因此，专家组认为，FISH是目前MM的主要遗传学检测技术，如条件允许可以进行二代测序检测，上述遗传学检测均需要进行浆细胞的富集分选。

2. 非遗传学高危因素：目前比较肯定的非遗传学预后因素，包括国际分期系统（ISS）Ⅲ期、非骨旁髓外病变、循环浆细胞、高浆细胞增殖指数、LDH升高、衰弱、肾功能不全以及血小板减少等。

（二）MM的动态预后因素

1. 初始治疗的缓解持续时间是MM关键的动态预后因素：对于接受auto-HSCT并序贯维持治疗的患者，从开始治疗到复发/进展不足2年，属于HRMM；而未接受auto-HSCT的患者，从开始治疗到复发不足18个月，属于HRMM[5]。功能性高危是指诊断时无已知的遗传学异常等高危因素，但开始治疗后18个月内早期进展的MM患者[6]–[7]。

2. 初始治疗的缓解深度也是MM重要的动态预后因素：随着MM治疗的进展，越来越多的MM患者能够取得深度缓解，而是否获得微小残留病（MRD）阴性逐渐成为重要的动态预后因素[8]。同时实现骨髓和影像学MRD阴性的患者具有最佳的生存结局[9]–[10]。达到MRD阴性可以部分克服高危细胞遗传学带来的不良预后，因此获得MRD阴性对于高危MM患者尤为重要。持续动态MRD监测相较于单次MRD检测结果更具临床价值，持续12个月以上的MRD阴性能够转化为长期生存[11]–[12]。

（三）MM的疾病分期和危险度分层体系

目前已有多个经过验证的MM预后评估体系在临床实践中应用，其中，修订版国际分期系统（R-ISS）[13]为临床应用最为广泛的分期系统，其他常见的分期系统包括ISS[14]、二次修订版国际分期系统（R2-ISS）[15]、IMWG危险分层[16]、mSMART 3.0（将具有1个HRCAs、R-ISS Ⅲ期、高S期浆细胞、基因表达谱高危特征、双打击及三打击定义为高危）[3]、MASS（Mayo additive staging system）[17]分期等。中国医学科学院血液病医院团队提出了新的加权骨髓瘤预后评分系统MPSS（Myeloma prognostic score system），该系统纳入多种遗传学异常、ISS分期及血小板减少因素，相较于R-ISS、R2-ISS等模型，可对中国MM患者实现更好的预后分层[18]（表1）。专家委员会建议在治疗过程中，应动态评估患者危险度。

**表1:** 常用的多发性骨髓瘤疾病分期及危险度分层体系

分期	ISS	R-ISS	IMWG	MASS^a^	R2-ISS^b^	MPSS^c^
Ⅰ期	β_2_微球蛋白<3.5 mg/L；血清白蛋白≥3.5 g/dl	ISS Ⅰ期，细胞遗传学标危，同时LDH正常	ISS Ⅰ或Ⅱ期；年龄<55岁；无t（4;14）、del（17p）、1q获得	Ⅰ期：0分	低危：0分	Ⅰ期：0分
Ⅱ期	Ⅰ期和Ⅲ期之间	Ⅰ期和Ⅲ期之间	Ⅰ期和Ⅲ期之间	Ⅱ期：1分	低危～中危：0.5～1分	Ⅱ期：1分
Ⅲ期	β_2_微球蛋白≥5.5 mg/L	ISS Ⅲ期，同时伴有细胞遗传学异常[t（4;14）,t（14;16）,17p−]或LDH升高	ISS Ⅱ/Ⅲ期同时伴t（4;14）或者del（17p）	Ⅲ期：≥2分	中危～高危：1.5～2.5分	Ⅲ期：2～3分
Ⅳ期	–	–	–	–	高危：3～5分	Ⅳ期：4～7分

**注** ISS：国际分期系统；R-ISS：修订版国际分期系统；R2-ISS：二次修订版国际分期系统；IMWG：国际骨髓瘤工作组；MASS：梅奥分期系统；MPSS：骨髓瘤预后评分系统；^a^加权得分：高危IgH异位、1q21获得/扩增、17号染色体异常、ISS-Ⅲ期、LDH高为各1分；^b^加权得分：ISS-Ⅱ期（1分）、ISS-Ⅲ期（1.5分）、del（17p）（1分）、LDH高（1分）、t（4，14）（1分）、1q21获得/扩增（0.5分）；^c^加权得分：低血小板（2分）、LDH高（1分）、ISS-Ⅲ期（2分）、伴有t（4，14）、t（4，16）、del（17p）、1q21获得/扩增中1个高危细胞遗传学异常（1分）、伴有2个及以上高危细胞遗传学异常（2分）；–：无

专家共识：①目前的临床分期和危险度分层体系，可有效辅助临床医师识别HRMM，但仍存在不完善之处。新的预后因素（如髓外病变、循环浆细胞等）的加入，使得HRMM的定义不断演化。当前各种危险度分层系统存在差别，高危遗传学异常的界定也存在混乱。此外，目前已确定的高危预后因素可能会被新的治疗方式所克服。②专家委员会认为，具备如下任何一项的初诊MM可认为是HRMM：R-ISS Ⅲ期、非骨旁髓外病变、存在循环浆细胞（外周血浆细胞比例≥5％，定义为浆细胞白血病）、具有1种及以上HRCAs［t（4;14）、t（14;16）、t（14;20）、del（17/17p）、1q21获得/扩增、del（1p32）、TP53突变］，但单独的1q21获得不能定义为HRMM；接受auto-HSCT并序贯维持治疗MM患者，从开始治疗到复发不足2年；未接受auto-HSCT患者，从开始治疗到复发不足18个月；功能性高危；髓外复发/继发浆细胞白血病；复发时新出现1q21获得/扩增和（或）del（17/17p）/TP53突变。

三、HRMM的治疗

（一）初治HRMM的治疗原则

由于缺乏HRMM的针对性临床试验，并且不同临床试验中HRMM的定义存在差别，因此HRMM标准治疗尚未确定。专家委员会认为，HRMM的总体治疗策略包括：①接受不同作用机制的药物联合治疗；②HRMM治疗的目标是根除所有肿瘤克隆，尽可能获得并维持骨髓内外MRD阴性；③HRMM应该采取根据疗效调整的治疗策略；④HRMM目前治疗效果尚不满意，鼓励探索试验性疗法。

（二）适合移植的初治HRMM的治疗

1. 移植前的诱导治疗：诱导治疗对于HRMM的价值不仅仅在于降低肿瘤负荷，同时应该考虑其获得MRD阴性的潜力。蛋白酶体抑制剂联合免疫调节剂及地塞米松的三药联合方案已经成为MM一线治疗方案。目前常用诱导治疗方案为RVd（硼替佐米+来那度胺+地塞米松）方案。研究证明HRMM接受RVd方案诱导桥接auto-HSCT，缓解深度及长期预后获益均不及预期。HRMM相较于标危患者较难获得MRD阴性，有必要采用新药改良方案加深患者缓解深度，改善HRMM患者预后[19]–[20]。FORTE研究[21]提示，具有1个HRCAs的患者接受KRd（卡非佐米+来那度胺+地塞米松）方案序贯auto-HSCT治疗的MRD阴性率及PFS与标危患者差异无统计学意义。另一方面，荟萃分析显示在早线治疗中加入CD38单克隆抗体作为骨架治疗的一部分，可实现HRCAs患者的临床获益[22]。对于UHRMM患者可以考虑更强的治疗方案，包括Dara-VRdC方案（OPTIMUM/MUKnine研究[23]）和Dara+KTD-PACE方案（TT7研究[24]）。

2. auto-HSCT：作为MM整体治疗的一部分，auto-HSCT有助于加深治疗深度并获得MRD阴性。串联移植（Tandem transplantation）是指在第1次auto-HSCT后的3～6个月内进行计划中的第2次auto-HSCT。HRMM可能从串联移植中获益（EMN02研究[25]和STaMIN研究[26]）。建议HRMM患者在第1次动员后尽量采集满足2次auto-HSCT所需的造血干细胞数量，第1次移植后无论获得何种疗效均建议在半年内进行串联移植。2次移植采用的预处理方案通常为大剂量美法仑（表2）。

**表2:** 适合移植的初治高危多发性骨髓瘤的关键临床研究结果

研究名称	方案	HR定义	HR比例（%）	MRD阴性率（阈值10^−5^，%）	中位PFS期/PFS率	OS率（%）
DETERMINATION（Ⅲ期）[20]	VRd+auto-HSCT对VRd	del（17p）、t（4;14）、t（14;16）	66/357（19.4）对66/365（19.8）	移植巩固治疗后患者：54.0对40.0（*P*>0.05）	55.5个月对17.1个月（*P*<0.05）	5年：63.4对54.3（*P*>0.05）
FORTE（Ⅱ期）[21]	KRd+auto-HSCT对KCd+auto-HSCT	del（17p）、t（4;14）、t（14;16）、del（1p）、1q获得/扩增	80/132（61.0）对92/138（67.0）	移植巩固治疗后1HRCA：69.0对53.0；2+HRCA：44.0对39.0	4年PFS率： 1HRCA：67%对55% 2+HRCA：55%对33%	4年：1HRCA：87.0对78.0；2+HRCA：74.0对55.0
Master（Ⅱ期）[22]	KRd-D+auto-HSCT	del（17p）、t（4;14）、t（14;16）、del（1p）和1q获得/扩增	70/123（57.0）	移植巩固治疗后1HRCA：86.0；2+HRCA：79.0	3年PFS率： 1HRCA：79% 2+HRCA：50%	3年： 1HRCA：92.0； 2+HRCA：75.0
GMMG-CONCEPT（Ⅱ期）[27]	KRd-Isa+auto-HSCT	del（17p）、t（4;14）、t（14;16）、1q扩增和ISS Ⅱ～Ⅲ期	99/99（100）	维持治疗后最佳缓解时HR患者：81.8	3年PFS率68.9%	3年：72.8
GRIFFIN（Ⅱ期）[28]	VRd-D+auto-HSCT对VRd+auto-HSCT	del（17p）、t（4;14）、t（14;16）	16/98（16.0）对14/97（14.0）	维持治疗后最佳缓解时HR患者：43.8对28.6（*P*>0.05）	随访49.6个月，HR患者：未达到对36.1个月（*P*>0.05）	未评估
PERSEUS（Ⅲ期）[29]	VRd-D+auto-HSCT对VRd+auto-HSCT	del（17p）、t（4;14）、t（14;16）	76/355（21.4）对78/354（22.0）	维持治疗后最佳缓解时HR患者：68.4对47.4	随访47.5个月，HR患者：未达到对44.1个月（*P*<0.05）	未评估
GMMG-HD7（Ⅲ期）[30]	VRd-Isa+auto-HSCT对VRd+auto-HSCT	del（17p）、t（4;14）、t（14;16）、1q21获得（≥ 3 copies）	125/331（37.7）对139/329（42.2）	诱导治疗后HR患者：50.4对37.3	未评估	未评估

**注** HR：高危；MRD：微小残留病；PFS：无进展生存；OS：总生存；d：地塞米松；Dara：达雷妥尤单抗；K：卡非佐米；R：来那度胺；V：硼替佐米；Isa：艾沙妥昔单抗；T：沙利度胺；1HRCA：仅有1个高危细胞遗传学异常；2+HRCA：具有两个及以上高危细胞遗传学异常

3. 巩固治疗：如患者未进行串联移植，可采用原诱导方案继续巩固治疗2～4个疗程。

4. 维持治疗：对于HRMM患者，应考虑加入蛋白酶体抑制剂、免疫调节剂及CD38单抗联合的两药或三药维持治疗。建议持续维持治疗直到疾病进展或不耐受。

5. allo-HSCT：allo-HSCT长期疗效仍有待商榷，鉴于不一致的临床研究结果、尚不明确的移植物抗骨髓瘤免疫作用及MM治疗出现更多新的选择（单抗、双特异性抗体和CAR-T细胞治疗等），应仅在临床试验的背景下，在特定的高危患者中选择进行allo-HSCT。

专家共识：①诱导治疗：HRMM移植前诱导治疗方案推荐使用CD38单克隆抗体联合蛋白酶体抑制剂和免疫调节剂为基础的方案，推荐方案包括：Dara+KRd（达雷妥尤单抗+卡非佐米+来那度胺+地塞米松）方案；Isa+KRd（艾沙妥昔单抗+卡非佐米+来那度胺+地塞米松）方案；Dara+VRd（达雷妥尤单抗+硼替佐米+来那度胺+地塞米松）方案；Isa+VRd（艾沙妥昔单抗+硼替佐米+来那度胺+地塞米松）方案；不能接受四药患者，可选用KRd方案；对于有明显髓外侵犯（软组织或者外周血）患者，可在此基础上加用细胞毒性药物，必要时联合放疗。②auto-HSCT：早期auto-HSCT是HRMM的标准治疗；接受auto-HSCT且无明显不良反应的患者，推荐移植后半年内进行串联移植。③巩固治疗：对未行串联移植的患者，建议使用原诱导治疗方案继续巩固治疗2～4个疗程。④维持治疗：推荐蛋白酶体抑制剂、免疫调节剂以及CD38单抗联合的两药或三药方案进行维持治疗，建议治疗直到疾病进展或不耐受。⑤鼓励开展针对HRMM的临床研究，建议HRMM患者首选入组临床试验。

（三）不适合移植的初治HRMM的治疗

对于不适合移植MM患者较难仅根据危险度分层和疾病分期来选择初始治疗方案，需根据患者的体能状况评分（推荐IMWG GA评分[31]）为患者选择个体化治疗方案。对于体能状态良好和中等健康患者，推荐采用与适合移植患者相同的方案持续治疗。对于虚弱患者，VRd-lite（改良的硼替佐米+来那度胺+地塞米松）方案和DRd（达雷妥尤单抗+来那度胺+地塞米松）方案是目前最为常用的一线治疗方案，目前已有临床试验探讨CAR-T细胞治疗（CARTITUTE-5研究）和双特异性抗体（MajesTEC-7研究）在不适合移植初治MM中的应用。

专家共识：①推荐不适合移植患者先行老年体能状态评估；②对于体能状况良好和中等健康的高危患者，推荐与适合移植患者相同的方案持续治疗；③对于虚弱的高危患者，应根据耐受情况选择个体化治疗，推荐虚弱患者参加免疫疗法的临床研究。

（四）复发HRMM的治疗

对于功能性高危以及根据动态危险因素定义的HRMM患者，再诱导方案推荐选择新一代药物或不同作用机制药物的联合方案。多项临床研究表明，BCMA CAR-T细胞治疗在多线复发难治MM患者中展现出显著的疗效，整体有效率达85％，完全缓解率近50％[32]–[34]。虽然各个临床试验样本量均较小，但大多包括了高比例的具有高风险特征的复发难治MM患者，CAR-T细胞治疗的疗效可以在复发HRMM中维持。

专家共识：①复发HRMM患者推荐选择新一代药物或不同作用机制药物的联合方案。②鼓励复发HRMM患者参加CAR-T细胞治疗或双特异性抗体等免疫治疗临床研究。

三、总结

HRMM作为目前临床诊疗的难点，其定义不断演化，探索新的预后标志物将有助于更好地识别HRMM。对于HRMM的治疗，建议应用不同作用机制药物的多药联合治疗并桥接auto-HSCT，以获得深度而持久的MRD阴性为治疗目标，从而延长患者OS期。目前针对初诊HRMM的CAR-T细胞临床试验正在陆续开展，auto-HSCT联合CAR-T细胞治疗，能同时发挥两者的治疗优势。除了BCMA靶点外，GPRC5D、FcRH5等靶点的CAR-T细胞治疗和双特异性抗体都在复发难治MM中取得良好疗效。新型药物及包含免疫治疗在内的新诊治策略有望突破HRMM治疗的瓶颈。
